# Changing approaches to rectal prolapse repair in the elderly

**DOI:** 10.1093/gastro/got025

**Published:** 2013-10-11

**Authors:** Vitaliy Poylin, Rodney Bensley, Deborah Nagle

**Affiliations:** ^1^Colon and Rectal Surgery, Beth Israel Deaconess Medical Center, Boston, MA, USA and ^2^Department of Surgery, Beth Israel Deaconess Medical Center, Boston, MA USA

**Keywords:** rectal prolapse, minimally invasive surgery, elderly

## Abstract

**Aim:** The abdominal approach to rectal prolapse is associated with lower rates of recurrence but a higher chance of complications and has been traditionally reserved for younger patients. However, longer life expectancy and wider use of laparoscopic techniques necessitates another look at the abdominal approach in older patients.

**Methods:** This was a retrospective review of data from patients undergoing abdominal repair of rectal prolapse between 2005 and 2011.

**Results:** Forty-six abdominal repairs (laparoscopic or open suture rectopexy, sigmoidectomy and rectopexy and low anterior resection) were performed during the study period. Twenty-nine repairs (63%) were performed in patients under the age of 70 (average age 51) and 17 (37%) in patients older than 70 (average age 76; range 71–89). Most of the cases performed during the initial 3 years of the study were via laparotomy. However, in the last 4 years, the laparoscopic approach was used in 83% of younger patients and 69% of older patients. Average length of stay was 2.6 days for younger and 3.8 days for older patients. Both groups had similar rates of re-admission: 20% vs 23%. The rate of wound infection was higher in the younger patients (5% vs nil). However, rates of urinary tract infection, two instances (10%) vs four (30%), urinary retention, one instance (5%) vs two (15.4%), ileus, one instance (5%) vs two (15.4%) were higher in the older group.

**Conclusion:** Wider use of laparoscopy has precipitated a change in the approach to rectal prolapse in older patients. Although associated with a slightly higher rate of post-operative complications, the abdominal approach to rectal prolapse is feasible, safe and effective in patients older than 70 years.

## INTRODUCTION

Full-thickness rectal prolapse is a benign but distressing condition that leads to problems with bleeding, fecal incontinence and obstructed defecation[1–3]. Rectal prolapse can significantly affect a patient’s quality of life and therefore should be repaired whenever possible. Older women are the most commonly affected group. In the western world, improvements in socio-economic and health conditions have led to an increase in the susceptible older population, highlighting the need for durable surgical repair of rectal prolapses.

Two major approaches are currently used to address this issue. The perineal approach—which has been traditionally reserved for older and debilitated patients—has been reported to be a better tolerated procedure with a lower rate of complications. This procedure, however, comes with a price of higher recurrence rates and functional changes (urgency, frequency) [1, 3–5]. Abdominal approaches carry a lower rate of recurrence, but often entail a bigger surgery with a higher complication rate [1, 3, 4, 6, 7].

Minimally invasive surgery has been well documented to lead to quicker recovery, less pain, shorter hospital stays and a lower complication rate [2, 8–10]. The wider use of laparoscopic rectopexy, which has benefits of minimally invasive surgery yet similar outcomes to open rectopexy, has prompted questions as to whether the abdominal approach should be more widely used in older patients, to achieve both an easier recovery and a more durable repair [11, 12–15].

The objective of this study is to analyse changes in clinical practice and outcomes of rectal prolapse repair in elderly patients.

## MATERIALS AND METHODS

Rectal prolapse was defined as a full-thickness prolapse, observed by experienced surgeons during their initial evaluation. Patients who underwent abdominal repair of rectal prolapse between 2004 and 2011 were identified from a prospectively collected database maintained at the Division of Colon and Rectal Surgery at Beth Israel Deaconess Medical Center (BIDMC). A retrospective review of all patient data was performed. For comparison purposes, all patients were divided into two groups: older (more than 70 years old) and younger (less than 70 years old). Demographics, procedure details, and the post-operative course, including complications, were analysed. Information on constipation and incontinence before and after surgery was collected but not scored.

Statistical analysis: pre-operative characteristics and outcomes are reported as proportions of the sample and mean ± standard deviation (SD). Patient variables were compared utilizing univariate analysis. Categorical variables were analysed using Fisher's exact test. Continuous variables were compared using the Wilcoxon rank sum test. Statistical significance was defined as *P* < 0.05. All statistical tests were performed using STATA 12 software (StataCorp, College Station, TX, USA).

## RESULTS

**Fig. 1.**
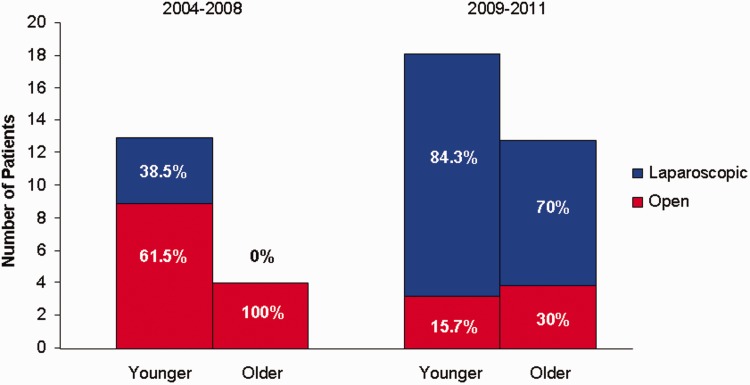
Trends in abdominal approach to rectal prolapse repair.

A total of 46 operations for rectal prolapse were performed during the study period. Operations performed were open and laparoscopic suture rectopexy, open and laparoscopic sigmoid colectomy with suture rectopexy, and one low anterior resection (one case early during study period). The decision on the type of surgery to be performed was made by the surgeon. A majority of patients who had sigmoid colectomy had associated constipation. During the initial part of the study period (2004–2008) most of the procedures in the younger group and all of the procedures in the older group were performed in the open fashion. In the later part of the study, between 2009 and 2011, the trend was reversed: most of the prolapse repairs in both the younger and older groups were performed laparoscopically (Fig. 1). Because laparoscopic rectal prolapse repair is quickly becoming a standard of care, in order to appropriately compare older and younger groups of patients, data from a later study period was used*.* All patients were entered into an accelerated recovery pathway utilized at our institution.

When comparing younger and older groups (Table 1), the latter group was significantly older [mean age 77 (71–90) vs 51.6 (24–69)], had a higher American Society of Anesthesia (ASA) class (2.75 vs 2.15) and a slightly lower body mass index (BMI) (24.3 vs 27.5).

**Table 1.** Patient demographics

	Younger	Older
**Age (years)**	51.6 ± 13.5	77.0 ± 5.3
**Female (%)**	90	100
**BMI (kg/m^2^)**	27.5	24.3
**ASA Class**	2.15	2.75

When comparing comorbidity profiles, the older patients had higher rates of congestive heart failure (23% vs nil, *P* = 0.05) as well as higher, but not statistically significant rates of coronary artery disease, chronic obstructive pulmonary disease, atrial fibrillation and inflammatory bowel disease. The rates of diabetes mellitus were higher, but not statistically significant in the younger group.

When comparing post-operative outcomes, older patients had higher rates of urinary tract infection [4 (30%) vs 2 (10%)] and higher rates of urinary retention, acute renal failure and ileus [2 (15.4%) vs 1 (5%)], but these did not reach statistical significance. Rates of wound infection and leakage were higher in the younger group [1 (5%) vs 0, with both complications in the same patient] (Table 2).

**Table 2.** Post-operative complications

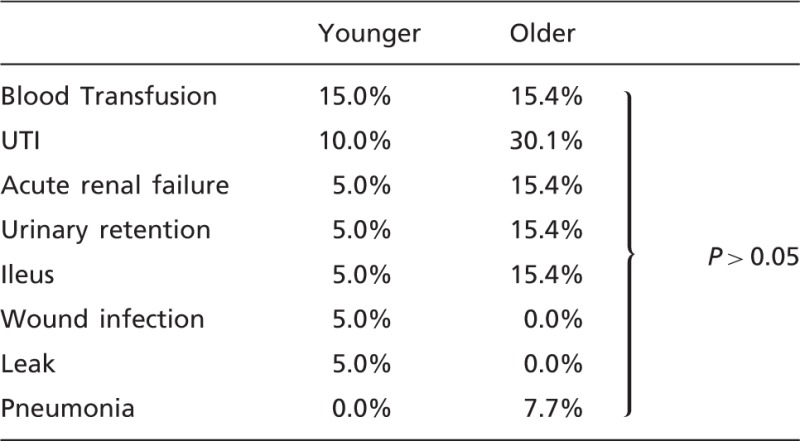

**Fig. 2.**
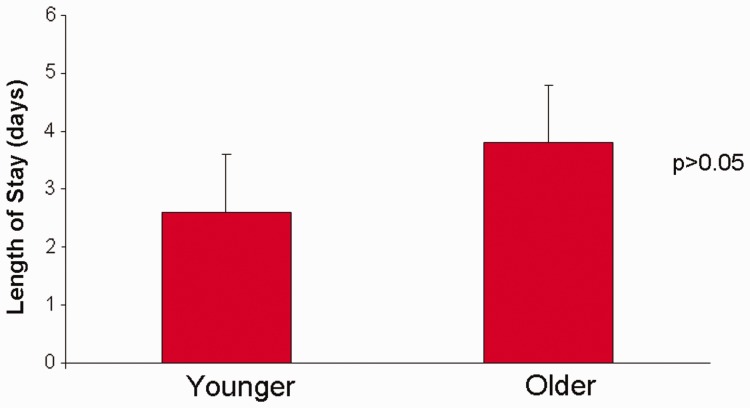
Post-operative length of stay.

The length of hospital stay was shorter in younger patients by more than a full day (2.6 + 1.0 vs 3.8 + 2.7), but it did not reach statistical significance (*P* = 0.09) (Fig. 2). All the younger patients—but only 77% of older patients—were discharged home. Rates of re-admission were similar in both groups (23% vs 25%). Rates of recurrence were higher in the older group (2 vs 1) (Table 3).

**Table 3.** Functional outcomes

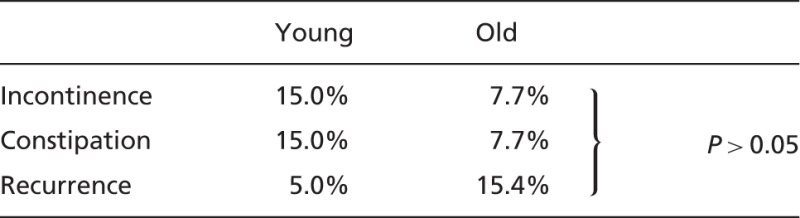

When subgroup analysis was performed comparing minimally invasive surgery and open approaches in younger and older groups, the results mirrored the overall data (Table 4), with recurrences higher in the laparoscopic group for both older and younger patients. Surprisingly, rates of urinary tract infections were higher in the laparoscopic group as well. Neither of these results reached statistical significance.

**Table 4.** Subgroup analysis of younger and older patients undergoing laparoscopic and open abdominal repair of rectal prolapse

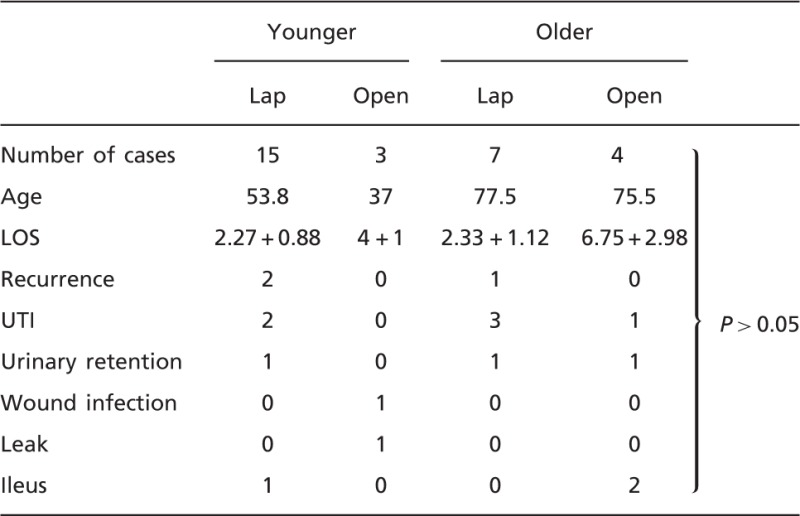

No formal surveys were administered to assess functional outcomes after abdominal repair. Patients were asked about rates of constipation and incontinence before and after the procedure. Although rates of both incontinence and constipation were higher in younger patients (15% vs 7.7%) these did not reach statistical significance. The average rates of follow-up were similar in both groups (average 6 months).

## DISCUSSION

Current studies have found that abdominal repairs of rectal prolapse are as safe and effective in older patients as they are in their younger counterparts. Traditionally, age was used as one of the major criteria to decide between the abdominal and perineal approach to repair of rectal prolapses: however, longer life expectancy and lower morbidity of laparoscopic techniques allowed for a closer look at the dogma.

In our study, the older patient group had more comorbidities, as defined by ASA classification, and individual conditions known to affect post-surgical outcomes. Older patients were also more debilitated, as evidenced by the higher numbers of those discharged into extended care facilities. As would be expected from more debilitated patients, older patients had both longer hospital stays (3.8 vs 2.6 days) and higher rates of most complications. Most of the complications reported were resolved with conservative treatment in both younger and older age groups.

Laparoscopy has been well documented to be a safe and effective technique for both younger and older patients [12]. Although the perineal approach is safe and successful for patients, the abdominal repair of rectal prolapse has been shown to be more durable [3, 6]. Especially for older patients, the balance between the morbidity of the procedure and overall outcomes must be carefully measured. As life expectancy is increasing in conjunction with advances in medical and surgical techniques, our traditional approaches need to be re-evaluated. In the case of rectal prolapse, emphasis has been shifting towards the more durable abdominal approach with better functional outcomes compared to the ‘safer’ (although also with significant associated morbidity) perineal approach. This and other recent studies show that recent changes towards the abdominal approach, specifically laparoscopic rectopexy (with and without sigmoid resection), make it a safe and effective way to repair rectal prolapse in elderly patients [14, 15]. Although most of the studies to date report somewhat higher rates of post-operative complications in abdominal surgeries, the rates are low enough to justify a more aggressive abdominal approach to prolapse.

In this study, all the recurrences took place in the laparoscopic group, which probably represents the learning curve associated with the new technique. In our study, rates of constipation and incontinence were similar for the two procedures (although higher in the younger group), indicating that older patients can expect a good functional result, both short- and long-term [14, 15]. No formal instruments were employed, however, in trying to assess functional outcomes for younger and older patients, which could introduce recall and recording bias.

The main problems with this study include its retrospective nature and small study population. There is also variability in the operations performed (with and without sigmoid resection), which could have influenced some of the outcomes. The rates of urinary complications reported in the older cohort were high and in the current environment of healthcare quality benchmarks, these complications represent an area where further investigation is needed to improve outcomes. Unexpectedly, rates of urinary tract infections were also higher in the laparoscopic group for reasons that are not clear. A prospective study may be needed to further evaluate the abdominal and minimally invasive approach to repair of rectal prolapse in elderly patients.

## CONCLUSION

The wider use of less-invasive laparoscopic techniques has precipitated a change in the approach to rectal prolapse in older patient cohorts. Although associated with slightly higher rates of immediate post-operative complications and length of stay, the abdominal approach to rectal prolapse repair may be feasible in select groups of patients older than 70 years.

**Conflict of interest:** none declared.
